# Transvenous pacing leads implanted in epicardial position: A recipe for future epicardial pacing electrode?

**DOI:** 10.1016/j.xjtc.2025.05.011

**Published:** 2025-05-28

**Authors:** Seraina Brütsch, Christian Balmer, Hitendu Dave

**Affiliations:** a Division of Congenital Cardiovascular Surgery, Pediatric Heart Centre & Children's Research Centre, University Children's Hospital Zürich, Zürich, Switzerland; b Division of Cardiology, Pediatric Heart Centre & Children's Research Centre, University Children's Hospital Zürich, Zürich, Switzerland

**Keywords:** congenital heart disease, epicardial pacing, pediatric, permanent pacemaker, transvenous 3830 selectsecure pacing lead

## Abstract

**Objective:**

Finding an optimum epicardial pacing site in children needing lead replacement can be challenging. We report the midterm outcome of using transvenous SelectSecure leads in the epicardial position.

**Methods:**

Between 2018 and 2020, 6 patients (5 children and 1 adult) received 8 SelectSecure 3830 leads (“off-label”) in the epicardial position: both the left atrium and left ventricle in 2 patients, only the left ventricle in 3 patients, and the right ventricle in 1 patient. The median age at lead implantation was 14 (4-35) years. Periodic pacing threshold, sensing, and impedance measurements were analyzed.

**Results:**

Lead implantations could be performed in all patients without complications, despite scarring from previous surgeries. The median pacing, sensing, and impedance measurements for 6 ventricular leads were 0.75 V, 6.5 mV, and 576 Ohm, respectively. The same for 2 atrial leads were 1.25 V, 2.5 mV, and 758 Ohm, respectively. During a follow-up period of 23.4 (8-32) months, despite a small increase at 6 months, pacing and sensing parameters remained acceptable. One reoperation occurred after the follow-up period due to lead dysfunction. One young patient with a complex structural heart disease and terminal heart failure on a ventricular assist device died while waiting for a heart transplant.

**Conclusions:**

The implantation of transvenous leads in the epicardial position is feasible and provides an alternative for cardiac pacing in patients with multiple previous surgeries and epicardial scarring. This lead design, although appealing, poses challenges for stable epicardial fixation. A larger experience and longer follow-up would decide its exact role in epicardial pacing.

**Figure:**
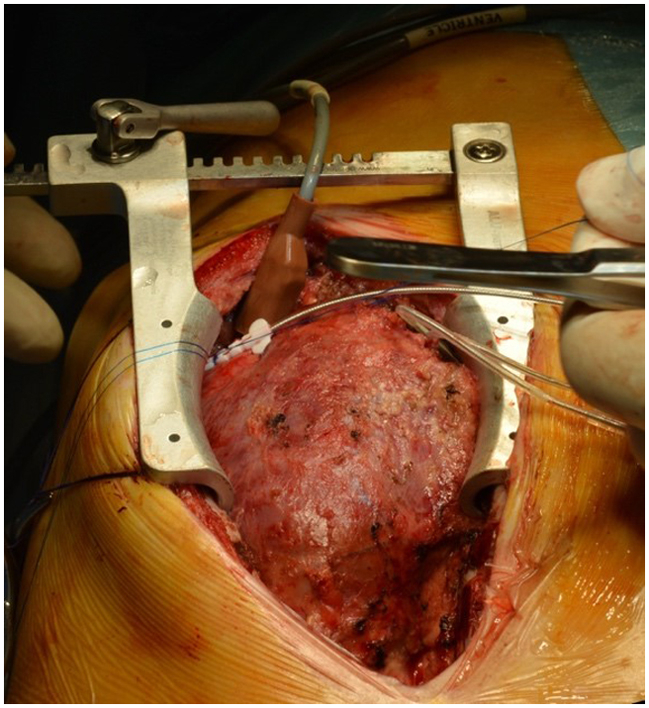
Intraoperative picture of a SelectSecure lead fixed to the heart.

**Central Message**

Transvenous SelectSecure lead implantation in the epicardial position is feasible. It may be considered in patients with multiple previous surgeries and pacing lead-induced scarring.

**Perspective**

This pilot study and the experience provide inputs for the design of an epicardial pacing electrode for the future.

Infants with congenital heart disease and congenital or postoperative conduction disorder often need a permanent pacemaker (PM) system within the first year of life, some requiring replacement within 2 to 5 years. The permanent PM leads can be attached to the heart in 2 ways: transvenous or epicardial.

The primary concern with transvenous placement of pacing leads in children is due to the small size of the veins. Transvenous PM implantation in young children is associated with a high incidence of vascular occlusion/thrombosis,^1^^,^^2^ severe regurgitation of atrioventricular (AV) valves, and endocarditis.^1^, ^2^, ^3^ Rapid body growth and increasing physical activity of the patients may lead to mechanical stress and lead dislocation of transvenous pacing systems. For these reasons, epicardial pacing systems are preferred for this age group.^4^ Conventionally, transvenous access was not considered an option for patients with Fontan circulation.^5^ However, innovative transcatheter access of the pulmonary venous atrium with placement of transvenous leads in Fontan patients has been described, although at the cost of lifelong anticoagulation and a potential risk of thromboembolism.^6^

Epicardial lead placement is still unequivocally considered more suitable for permanent pacing in infants and small children. Despite that, this approach has its downsides. This procedure is invasive (requiring subxiphoid approach, sternotomy, or thoracotomy) and can be technically more challenging.^7^^,^^8^ Epicardial leads also may be associated with a significant failure rate.^8^^,^^9^ In a prospective follow-up study, using bipolar steroid-eluting epicardial leads, it was shown that although no intraoperative complications occurred, several reoperations were necessary within 5 years, almost all because of lead problems. The main reason was lead fracture caused by muscular activity.^8^ Young children, who primarily benefit from the epicardial pacing system, also have a higher rate of reintervention.^5^^,^^7^^,^^10^, ^11^, ^12^ Reinterventions typically are required due to exhausted battery power or to lead dysfunction. The former occurs earlier in children because of higher energy consumption (due to higher heart rate); the latter is also more frequent in children because of greater physical activity and the intercostal course of the electrodes, and the exposure of the lead to PM interphase to harsh muscular activity.

Rapid physical growth in children leads to lead stretching. The leads rarely may develop insulation defect due to mechanical stress. Prolonged exposure of leads in the body may weaken the insulation, deposit calcification, resulting in de novo lead fracture, or may subject them to increased risk to injury during surgical manipulation. Suturing the leads to the surface of the heart induces local ischemia and eventually results in fibrotic scarring. The epicardium becomes thickened and is not electrically as conductive as a virgin myocardium. Consequently, most often another position must be found during reoperation. Finding an optimal location to achieve the best interventricular/intraventricular synchrony and achieving the lowest possible threshold in a reoperative environment can be challenging.

The Medtronic SelectSecure 3830 transvenous lead is a lumenless single strand of bipolar lead (with both poles placed at the tip ∼1 cm apart) and is described as magnetic resonance conditional when used in a classic transvenous situation. The lumenless linear design without bifurcating poles makes it thin and thus possibly less susceptible to lead dysfunction compared with other transvenous leads. This lead has been developed, tested, and certified for transvenous use (CE approval 31.01.2003; US market release 03.08.2005).

When confronted with a suboptimal position or high stimulation threshold, often in reoperative environments, we considered using the Medtronic SelectSecure 3830 transvenous lead in the epicardial position. Informed consent of the patient or parental caregivers was obtained. This retrospective study aims to analyze the feasibility, efficacy, and midterm longevity of the transvenous SelectSecure electrodes in the epicardial position. The study explores the hypothesis that this technique helps to quickly achieve a satisfactory stimulation threshold at a desired location, despite adhesions and epicardial scarring.

## Material and Methods

### Institutional Review Board Statement

The study design was conducted in accordance with the Declaration of Helsinki. The Cantonal Ethics Committee of Zürich, Switzerland, decided that no ethical approval was necessary for this case series (phone consultation and e-mail from Cantonal Ethics Committee of Zurich, dt. 21.04.2020). A written informed consent was obtained for all patients from the parental caregivers. All patient data were extracted from the medical record system of the Children's Hospital of Zurich and the University Hospital of Zurich.

### Inclusion Criteria

All SelectSecure implantations performed by a single surgeon (H.D.) at University Hospital and University Children's Hospital Zurich between 2018 and 2020 were included in the analysis.

### Patients

The study cohort consisted of 6 patients (5 children and 1 adult) with congenital heart disease, all being reoperations after repair of congenital cardiac defects or previous PM implantations. However, patient number 6 was a post-Fontan patient who received the SelectSecure electrode as the primary PM electrode, because we assumed that this electrode would serve the best long-term interest of the patient. Demographic characteristics are shown in Table 1.

**Table 1:** Demographic, anatomic, and electrophysiological characteristics

Characteristics	No. of patients
Male/female	4/2
Age at SelectSecure implantation, y	11 (4-35)
Age at follow-up, y	15.5 (7-38)
Median follow-up, mo	27 (8-32)
Cardiac anatomy	
Normal cardiac anatomy	1
AV septal defect	2
Double-inlet LV	2
Hypoplastic right heart syndrome	1
Indication for PM	
Congenital AV block	1
Postoperative AV block	3
Resynchronization therapy	1
Sinus node dysfunction	1

Values are shown as median (range) or number of patients. *AV,* Atrioventricular; *LV,* left ventricle; *PM,* pacemaker.

### Leads

**Figure 1:**
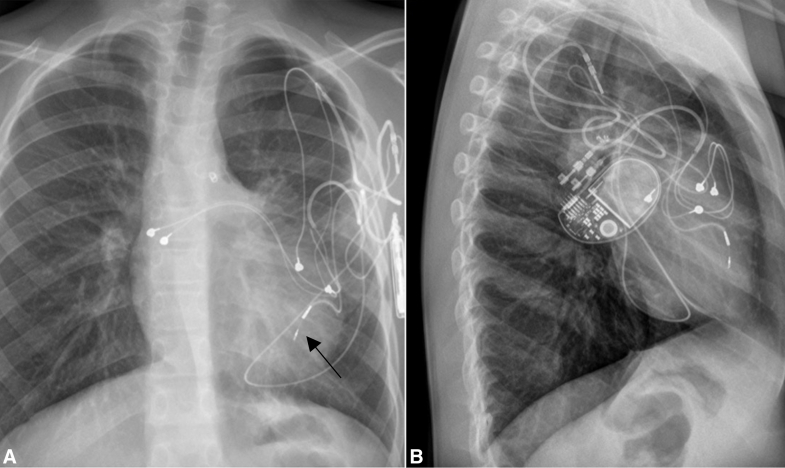
Chest x-ray images after SelectSecure lead implantation. Shown are transmyocardial position of SelectSecure MRI SureScan 3830 bipolar lead stimulating the LV (*black arrow*), conventional epicardial leads on the right atrium, and capped dysfunctional conventional epicardial lead in the left axillary extrathoracic pocket. A, Posterior-anterior view. B, Lateral view.

SelectSecure MRI SureScan 3830 leads (Medtronic) were implanted in the epicardial position. The leads were placed in the following anatomic locations: the left atrium and left ventricle (LV) in 2 patients, only the LV in 3 patients, and the right ventricle (RV) in 1 patient (Figure 1).

### Surgical Approach

**Figure 2:**
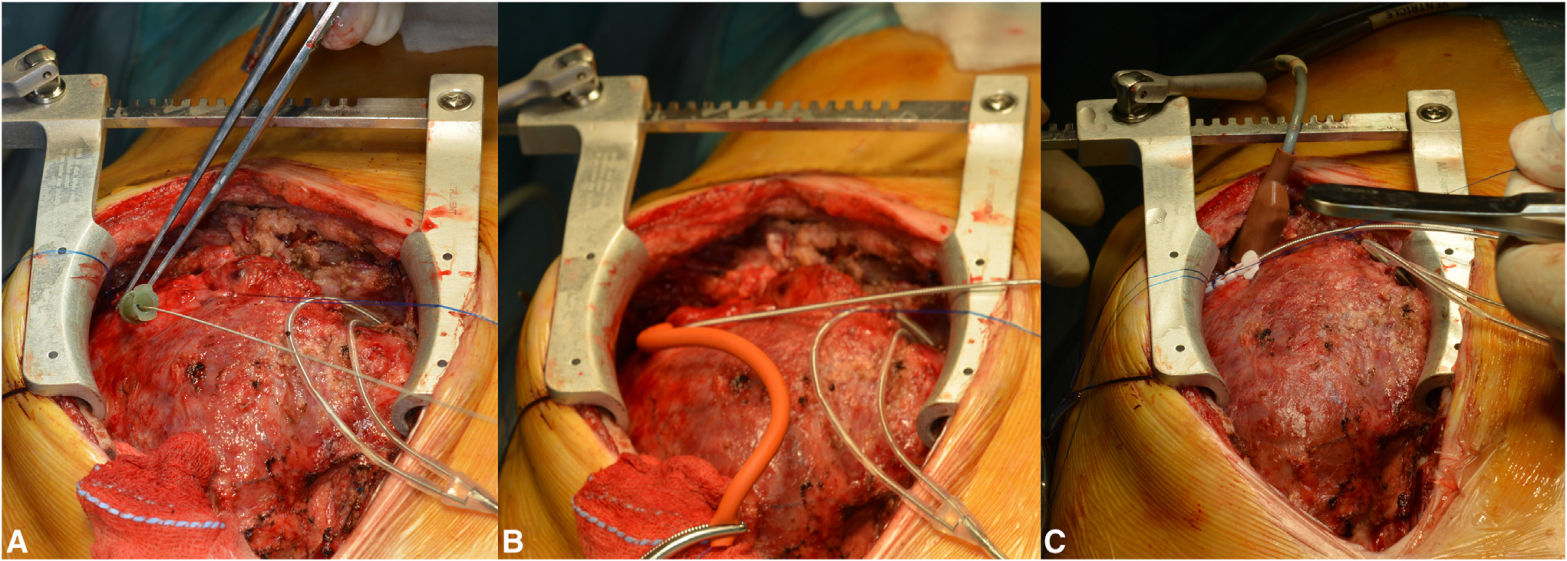
Stepwise procedure of implantation technique through a sternotomy. A, Epicardial insertion of the needle along with the guidewire at the desired location (LV apex in this case). B, Placement of the lead in the tunnel using screwing motion to ensure that both the poles of the bipolar lead are in contact with the myocardium. The lead is held in position with a tourniquet while electrophysiological measurements are performed. C, The lead is secured in position using the supplied plastic sheath at the epicardial entry site.

A lateral thoracotomy was chosen as the surgical approach in 3 patients and a median sternotomy in the remaining 3 patients. After clear identification of the cardiac structures and landmarks discerning the LV from the RV, an oblique intramuscular tunnel was created by using the Seldinger needle. The SelectSecure transvenous lead was inserted epicardially so that both the poles remain in contact with healthy myocardium. The provided plastic butterfly sleeve was passed into the entry site epicardium and secured with nonresorbable sutures (Figure 2).

### Definitions

The functionality of the leads was tested using the 3 conventional parameters.

• Pacing threshold (V)
• Sensing (mV)
• Impedance (Ohms)

The first measurements were obtained immediately after implantation and before discharge from the hospital. Follow-up measurements were performed at 1-, 3-, and 6-month intervals.

### Statistical Analysis

Data are presented as median (range) or mean (±SD). Statistical analysis was performed using Microsoft Excel 2022.

## Results

### Operations Before SelectSecure Implantation

As shown in Tables 2 and 3, all 6 patients had undergone surgical procedures before the index SelectSecure implantation procedure. Of these, 4 had undergone 7 PM-related operations at a median (range) duration of 8 (3.5-10) exposure years after PM implantation. The remaining 2 patients had various cardiac surgical procedures related to congenital heart diseases. The probability of PM-related reoperation was 0.2 per patient-year. Indications for previous PM-related procedures included battery depletion (n = 2), infection (n = 2), and lead dysfunction (n = 3). All the previous PM systems involved standard bipolar steroid-eluting leads.

**Table 2:** Reoperations before SelectSecure implantation

Patient serial no.	No. of PM unrelated reoperations before SelectSecure implantation	No. of electrode-related reoperations before SelectSecure implantation	Exposure to PM (y) before SelectSecure implantation	Sternotomy/thoracotomy approach for SelectSecure implantation
1	0	0	10	Thoracotomy
2	0	2	10	Thoracotomy
3	0	1	3.5	Sternotomy
4	1	0	2	Thoracotomy
5	0	0	6	Sternotomy
6	1	0	0	Sternotomy
Total procedures before SelectSecure implantation	2	3		
Total PM exposure (y) before SelectSecure implantation			31.5	

All patients have had at least 1 previous operation. One patient (serial No. 6) did not have a PM before SelectSecure implantation. In this case, epicardial implantation of the SelectSecure lead was chosen directly due to previous repeated cardiac interventions and corresponding scarring. Transvenous implantation was not considered due to Fontan physiology.

*PM,* Pacemaker.

**Table 3:** Measured values of the SelectSecure leads (n = 8) over time, stratified by atrial or ventricular lead location

Interval (mo)		0	6	12	18	24
Atrial leads (n = 2)	Pacing threshold at 0.4 ms (V)	1.25 (0.5-2)	1.75 (1-2.5)	1.75 (1-2.5)	2.38 (0.75-4)	2.25 (0.75-3.75)
	Sensing (mV)	2.5 (2-2.9)	2.8 (2.5-3)	2.6 (1.5-3.8)	1.6 (1.3-1.8)	5.5 (1.6-9.3)
	Lead impedance (Ohm)	758 (722-793)	456	466 (456-475)	551 (513-589)	532 (475-589)
Ventricular leads (n = 6)	Pacing threshold at 0.4 ms (V)	0.75 (0.5-1.5)	1.19 (1-2.5)	1.75 (1-2)	1.5 (0.5-2.25)	1.63 (0.75-1.75)
	Sensing (mV)	6.5 (2.5-18.5)	10 (4-11)	7.5 (5.3-12.5)	11.3 (7-20)	11.3 (6-20)
	Lead impedance (Ohm)	576 (513-2185)	546 (513-2166)	551 (530-2109)	561 (490-1976)	541 (513-1995)

Values are presented as median (range).

### Surgery

During the index procedure, a standard steroid-eluting bipolar lead was tried in 1 patient before turning to the SelectSecure lead; in the remaining patients, the local findings of scarring at the preferred site prompted us to use the latter directly. In all 6 patients, the SelectSecure lead (Medtronic) implantation could be performed successfully in 2 atrial and 6 ventricular locations. One difficulty (which may be described as part of the learning curve) was to find the right plane underneath the thickened epicardium, but within the myocardium to place the lead (Figure 2, *A* and *B*). We did not aim to position the tip of the electrode into the ventricular lumen because of the variable thickness of the LV apical septum. Thus, transesophageal echocardiography was available but not systematically used to track the lead position. However, this is an option if we consider an intraluminal position of the lead. The biggest challenge of course was to ensure a robust fixation over the heart, which was improvised by using the plastic sleeve around the lead (Figure 2, *C*). Despite scarring and adhesions due to previous operations, the intramyocardial SelectSecure strategy helped to quickly achieve good thresholds at desired locations. No perioperative complications were recorded.

### Lead Function Over Time

**Figure 3:**
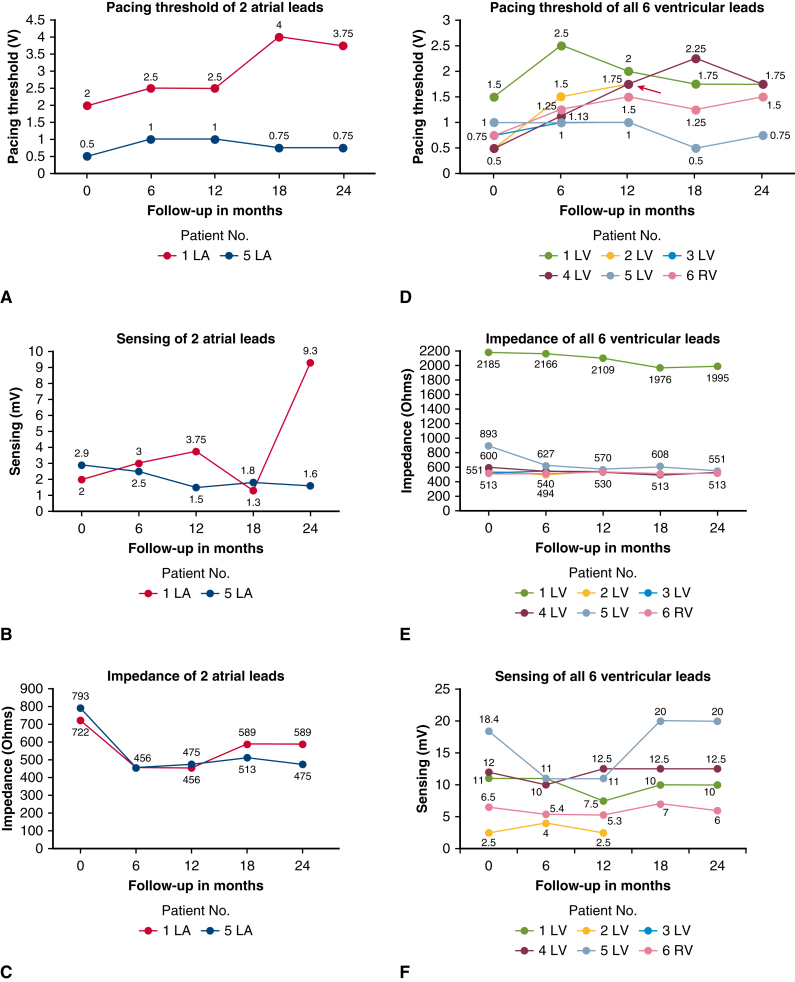
Detailed longitudinal values of the SelectSecure leads (n = 8) over time. A-C, Pacing, sensing, and impedance measurements of 2 leads in the atrial position. D-F, Pacing, impedance, and sensing measurements of 6 leads in the ventricular position. Patient number 2 (shown by *red arrow* in D) needed lead replacement due to lead dysfunction approximately 1 year postimplantation. *LA,* Left atrium; *LV,* left ventricle; *RV,* right ventricle.

The immediate postimplantation pacing threshold of 1.25 V for atrial leads and 1 V for ventricular leads was satisfactory (Table 3). The initial atrial wave sensing of 2.5 mV, and the ventricular wave sensing of 9.9 mV was adequate. During a median follow-up of 27 months (range, 8-32 months), the atrial pacing threshold increased slightly to 2.25 V and the sensing and impedance remained stable. An overview of the longitudinal course of all measurements is shown in Tables 3 and 4 and Figure 3. Patient number 2 (marked red in Figure 3) developed ventricular lead dysfunction 19.6 months after implantation, which manifested as increased threshold and unstable sensing measurements. This patient received lead replacement using a conventional steroid-eluting bipolar lead. Since this early reoperation, we have set a higher threshold to using the SelectSecure lead, notwithstanding the fact that we would consider it an option if a conventional lead does not deliver good results. The Kaplan–Meier freedom from PM/PM lead reoperation was 80% (20.4%-86.9%) at 24 months.

**Table 4:** Measured values of the SelectSecure leads (n = 8) over time, broken down by patient

Serial no.	Lead	Interval (mo)	0	6	12	18	24
1	1A	Pacing threshold at 0.4 ms (V)	2	2.5	2.5	4	3.75
		Sensing (mV)	2	3	3.8	1.3	9.3
		Lead impedance (Ohm)	722	456	456	589	589
	1V	Pacing threshold at 0.4 ms (V)	1.5	2.5	2	1.75	1.75
		Sensing (mV)	n/a	11	7.5	10	10
		Lead impedance (Ohm)	2185	2166	2109	1976	1995
2	2V	Pacing threshold at 0.4 ms (V)	0.5	1.5	1.75		
		Sensing (mV)	2.5	4	2.5		
		Lead impedance (Ohm)	551	494	551		
3	3V	Pacing threshold at 0.4 ms (V)	0.75	1			
		Sensing (mV)	n/a	n/a			
		Lead impedance (Ohm)	513	551			
4	4V	Pacing threshold at 0.4 ms (V)	0.5	1.13	1.75	2.25	1.75
		Sensing (mV)	12	10	12.5	12.5	12.5
		Lead impedance (Ohm)	600	540	530	490	530
5	5A	Pacing threshold at 0.4 ms (V)	0.5	1	1	0.75	0.75
		Sensing (mV)	2.9	2.5	1.5	1.8	1.6
		Lead impedance (Ohm)	793	456	475	513	475
	5V	Pacing threshold at 0.4 ms (V)	1	1	1	0.5	0.75
		Sensing (mV)	18.4	11	11	20	20
		Lead impedance (Ohm)	893	627	570	608	551
6	6V	Pacing threshold at 0.4 ms (V)	0.75	1.25	1.5	1.25	1.5
		Sensing (mV)	6.5	5.4	5.3	7	6
		Lead impedance (Ohm)	513	513	532	513	513

### Late Mortality

One child underwent Berlin Heart biventricular assist device therapy leading to the removal of the SelectSecure electrodes 7.9 months postimplantation. This patient died during the 9.3 months after Berlin Heart Bi-VAD therapy. This death was not associated with the lead or the PM system. The child with dilatative cardiomyopathy consequent to multiple operations for complex congenital structural heart disease received an LV and RV lead for cardiac resynchronization therapy. Being supported on a Berlin heart as a bridge to heart transplantation, he had a fatal hemorrhagic stroke.

## Discussion

Although few patients with an AV block recover while on PM therapy, most children need pacing lifelong for high-grade AV block, sinus node dysfunction, or cardiac resynchronization. This in turn means that multiple PM procedures in addition to change of battery are preprogrammed in a lifetime. The product development for children traditionally has been a leftover from the adult domain, and PM therapy is no exception. The conventional epicardial PM electrodes 4968 CapSure Epi (Medtronic) in use today have been developed and certified for marking in 1990s. The difficulty in finding a location with low pacing threshold with a goal of pacing the LV apex to preserve interventricular synchrony means that epicardial pacing sites during reoperations can be challenging to find. In pursuit of quickly achieving these goals, we decided to use the SelectSecure leads in dire conditions. Our pilot study experience in 6 patients showed the feasibility and ease of implantation of these electrodes with good pacing and sensing values, as well as resistances known with conventional electrodes. The good values were achieved quickly. Although the pacing threshold remained stable in most cases, there was a slight increase in 2 patients, 1 of whom required a reoperation within 19.6 months. These undulating values seen in few patients may be due to the difficulty in stable fixation of these electrodes. Because this lead design is not meant for epicardial fixation, the cardiac motion remains a challenge.

Although 2 of the 6 patients in our cohort were aged 16 and 35 years (with Fontan physiology), the remaining patients were aged 10 years and younger. Although we cannot advise on the optimum age for considering this type of electrode, because of our positive experience with epicardial pacing, we do believe in the epicardial system in children who have still not achieved their fully grown stature. We also continue to believe in LV pacing, which is best achieved by an epicardial approach.

Yuan and colleagues^13^ reported a successful case of using the Medtronic 3830 electrode in a 2-year-old child with iatrogenic third-degree AV block after the Ross-Konno procedure. Although the procedure is similar to ours, there are no details on the way the electrode was fixed epicardially or on the follow-up.

Sandrio and colleagues^14^ reported their experience with transmural placement of endocardial pacing leads in patients with congenital heart disease. Although most leads were placed under direct observation with concomitant intracardiac repair, the authors documented an incidence of 33% ventricular lead dysfunction/dislocation during a median follow-up period of 2 years. They report a Kaplan–Meier freedom from lead dysfunction of 73% ± 13% at 1 year and stable ventricular threshold of 0.6 ± 0.3 V in the atrial position and 0.9 ± 0.5 V in the ventricular position. The overall experience in literature with this strategy is so limited that a direct comparison is not possible. However, these values need to be compared with the outcome of conventional epicardial pacing leads in similar challenging situations. Aellig and colleagues^11^ performed a long-term follow-up study after PM implantation in infants using conventional epicardial leads at the Children's Hospital of Zurich in 2007. Comparing these measurements with those of our study, both ventricular pacing thresholds and atrial sensing values were broadly comparable, although the 6-month follow-up values tended to be relatively higher with SelectSecure leads. However, the present cohort of 6 patients with a short follow-up duration does not allow us to draw any definitive conclusions. Wildbolz and colleagues^15^ showed excellent results of epicardial pacing in neonates and infants. Winkler and colleagues,^16^ from our group, reported epicardial lead survival of 75.59% (70.11%-80.21%) at 10 years and 50.07% (38.9%-60.24%) at 15 years in a cohort of 238 children with a follow-up of 1998 patient-years.

Notwithstanding the difficulty in fixation and stabilization of the SelectSecure leads, this study also was meant to assess the advantages of this bipolar single-strand design. The single-strand design compared with the Y-shaped design of the conventional epicardial electrodes makes entrapment in adhesions less cumbersome, with potentially less chance of coronary strangulation and ease in removal if that becomes necessary. The ease of finding an optimum pacing site due to the transmural stimulation of untouched myocardium is a unique advantage offered by this design. It is also conceivable that by evolving a technique of transmural puncture into the LV apex so that the tip stimulates the trabecular endocardium and the ring, the muscular myocardium may offer better values and stability. Our experience aims to offer inputs to incorporate these ideas in the design of a future epicardial pacing electrode.

### Limitations

The study was performed under “off-label use' because this lead has not yet been certified for epicardial implantation. This also explains the small study population. Furthermore, the study was not a randomized case-controlled study. The short follow-up duration of 2.6 years means that the long-term outcomes are not yet known, and no conclusions can be drawn about the equivalence or superiority of these electrodes.

## Conclusions

Our short-term pilot study with the implantation of transvenous SelectSecure leads in the epicardial position demonstrates the safety and feasibility of this procedure. It appears to be a valid alternative when conventional electrodes are not expected to achieve desirable pacing and sensing thresholds due to a hostile surgical environment. The design and development of the SelectSecure lead are inherently not optimal for epicardial implantation and attachment to the cardiac surface. However, the concept of transmural pacing through a single bipolar lead is attractive. These early encouraging results should serve as a basis for further research in this field and enable development of better solutions for our young patients in need of lifelong pacing.

## Conflict of Interest Statement

The authors reported no conflicts of interest.

The *Journal* policy requires editors and reviewers to disclose conflicts of interest and to decline handling or reviewing manuscripts for which they may have a conflict of interest. The editors and reviewers of this article have no conflicts of interest.
